# Comparative Impacts of Freight and Non-truck Traffic on NO_*x*_ and Ozone Concentrations in the Los Angeles Basin

**DOI:** 10.1021/acsestair.5c00396

**Published:** 2026-01-21

**Authors:** Aryiana C. Moore, T. Nash Skipper, Armistead G. Russell, Jennifer Kaiser

**Affiliations:** † School of Civil and Environmental Engineering, 1372 Georgia Institute of Technology, Atlanta, Georgia 30332, United States; ‡ School of Earth and Atmospheric Sciences, Georgia Institute of Technology, Atlanta, Georgia 30332, United States

**Keywords:** machine learning, random forest, ozone production regime, freight activity, air quality

## Abstract

The Los Angeles (LA) metropolitan region remains in nonattainment for ozone despite decades of reductions of ozone precursors, nitrogen oxides (NO_*x*_) and volatile organic compounds (VOCs). NO_*x*_ emissions from freight vehicles (ships, heavy duty trucks, trains, and airplanes) are expected to exceed emissions from passenger vehicles in southern California by 2030. Here, we use random forest machine learning to estimate the impact of freight activity on hourly NO_*x*_ concentrations and determine summertime ozone production regimes across the LA basin. We find that freight activity contributes over half of weekday NO_*x*_ impacts relative to non-truck traffic. During peak ozone hours, coastal areas, south LA, areas downwind (east) of downtown LA, and downtown San Bernardino are VOC-limited. Our results suggest that as of 2021, the Los Angeles urban core and nearby downwind areas have not transitioned to a NO_*x*_-limited regime on most days during the May to September ozone season. This study shows the applicability of machine learning to estimate concentration impacts from specific sources in the face of uncertain emission inventories and to analyze current ozone production regimes in areas with hourly ground observations.

## Graphical abstract

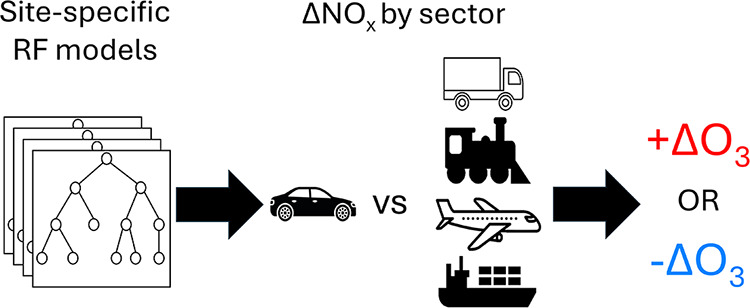

## 1 Introduction

Despite decades of reductions in ozone precursors, nitrogen oxides (NO_*x*_ = NO + NO_2_) and volatile organic compounds (VOCs), the Los Angeles (LA) basin remains in nonattainment for ozone.^1^ The sensitivity of ozone concentrations depends on the prevailing chemical regime: under NO_*x*_-limited conditions, ozone production is more sensitive to changes in NO_*x*_ emissions, whereas under VOC-limited conditions, ozone production is more sensitive to changes in VOC emissions. Additionally, in a VOC-limited regime decreased NO_*x*_ emissions may increase ozone concentrations. Much of the LA basin has historically been in a VOC-limited regime, but given the stall in ozone reductions in recent years,^2^ there has been a renewed focus on understanding the remaining NO_*x*_ and VOC emission sources and determining whether the ozone production regime has shifted.

Chemical transport model (CTM)-based studies analyzing maximum daily 8-h average (MDA8) ozone concentrations have concluded that areas outside of LA’s urban core are now in the NO_*x*_-limited regime or are nearing the transition point.^2,3^ These modeled results are supported by decreased MDA8 ozone weekday/weekend effect^2,3^ and empirically derived isopleths based on ozone design values.^4^ A box model analysis indicates ozone production in Pasadena, which is downwind of LA, is VOC-limited during peak ozone hours but may become NO_*x*_-limited in afternoon hours.^5^ In contrast, CTM-based studies with updated VOC emissions conclude LA’s urban core and downwind areas still need further NO_*x*_ reductions before reaching NO_*x*_-limited conditions.^6,7^ COVID lockdown studies^8,9^ and chamber perturbation experiments^10^ also suggest areas downwind of downtown Los Angeles are VOC-limited.

Complicating future emission control strategies, the relative magnitude of NO_*x*_ emission sources in the region is expected to shift dramatically in the near future. The California Air Resources Board (CARB) predicts that absent federal regulation NO_*x*_ emissions from ships, interstate trucks, trains, and airplanes will exceed emissions from passenger vehicles and intrastate trucks in southern California by 2030.^11^ However, previous studies suggest NO_*x*_ emissions attributable to freight transit in LA may be underestimated in traditional emission inventories meaning these federally regulated freight sectors may have a higher impact than currently estimated.^12,13^ Aircraft-observed NO_*x*_ fluxes suggest that the 2020 CARB emission inventory likely underestimates freight-related NO_*x*_ emissions over San Bernardino due to unaccounted for increases in diesel vehicles caused by increased warehouse activity in the area.^12–15^ While Los Angeles International Airport (LAX) and Ontario International Airport (ONT) are identified as major contributors of NO_*x*_ emissions by CARB,^11,13^ uncertainties in airport emissions from traditional inventory modeling are known to increase uncertainties in modeled air quality and health impacts.^16–19^ The San Pedro Bay ports, Port of Los Angeles (POLA) and Port of Long Beach (POLB), impact NO_*x*_ concentrations near Long Beach via emissions of freight-associated locomotives, heavy-duty vehicles, ocean vessels, and cargo handling equipment, but emission inventories may not accurately capture total port emissions.^13,20,21^

Previous studies show that machine learning (ML) methods have high predictive accuracy, performing better or equal to traditional CTMs while avoiding problems with emission inventory uncertainty.^22–25^ The large change in activity patterns during the COVID-19 lockdown has provided a test of the ability of ML techniques to separate the impact of emission changes from meteorological influences on air pollution.^26–28^ Two techniques are commonly used. In the first, a ML model is trained using data before COVID. Then pollutant concentrations are predicted for the COVID-19 date range using the trained model, representing a business-as-usual scenario (BAU). The observed pollutant concentrations are then subtracted from the BAU scenario to give COVID-19 impacts.^26,27,29^ The second method is to train the model using the entire data set and use the model to understand the importance of meteorological features vs emission-based features.^30^ While most COVID-19 ML studies generalize air quality changes broadly as a reduction in anthropogenic activities,^26–30^ some attribute impacts to specific sectors such as vehicle traffic or airport activity.^31,32^ However, the limitations of ML interpretability and utility for sensitivity analyses require further evaluation.^33,34^

Here, we use random forest (RF) models trained using ambient ground monitors and freight activity data to estimate the impact of freight transit on ozone and NO_*x*_ across Los Angeles from 2018–2021. We compare the relative contributions to observed NO_*x*_ concentrations from freight (i.e., airport, rail, seaport, and truck) activity and non-truck traffic activity. Lastly, we assess the capability of RF models to determine ozone production regimes and compare our results to a weekday/weekend analysis.

## 2 Methods

### 2.1 Ambient Observations

We trained RF models to predict ozone, NO_*x*_, and O_*x*_ (NO_2_ + ozone) concentrations measured at ∼30 monitors. O_*x*_ is conserved under high-NO scavenging conditions (NO + O_3_ → NO_2_ + O_2_) and is a useful parameter for evaluating ozone budgets in polluted regions. Each site-specific RF model was trained using hourly ground observations for weekdays during January 2018 to December 2021. Ground observations were collected from monitors in the California Ambient Air Monitoring Network.^35^ Of the active CARB sites, 25 sites were used for NO_*x*_, 29 sites for ozone, and 24 sites for O_*x*_ (Figure 1 and Table S1). Of the 25 NO_*x*_ sites, 4 are near road monitors. No near road monitors were used for modeling ozone or O_*x*_. Concentration data from monitors was not filled or interpolated for use in models.

**Figure 1:**
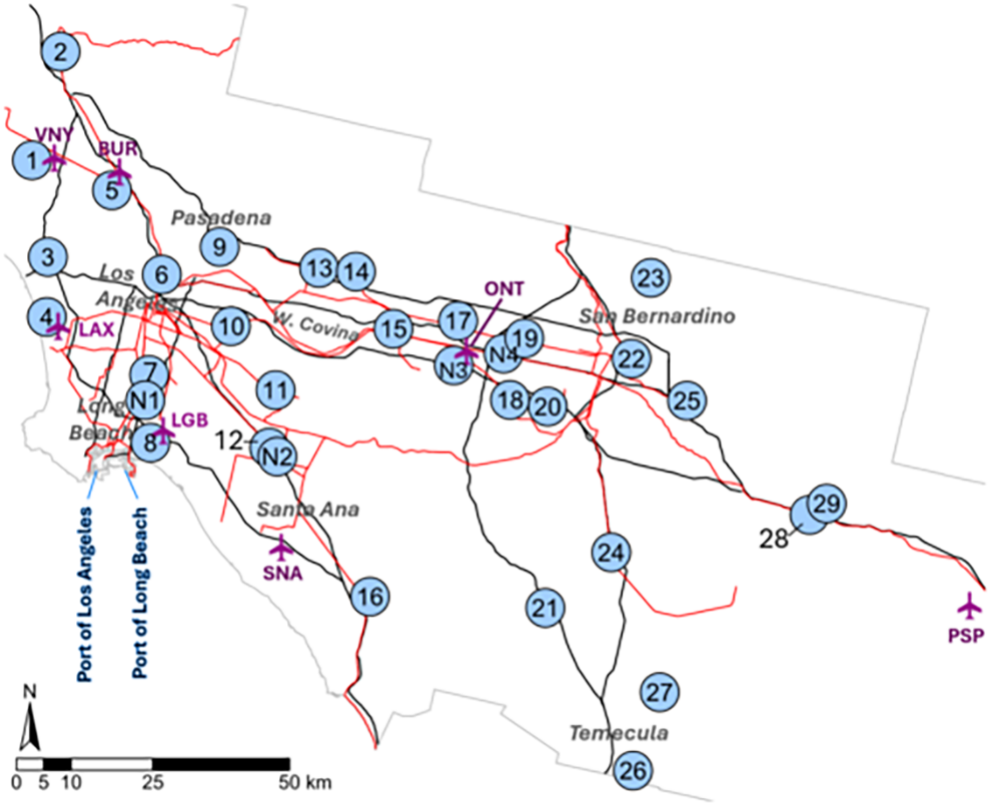
Map of CARB ground monitor network used for training RF models. Monitors are numbered from west to east. Near road monitors are numbered with an N preceding. Major highways (black), freight allowed rail lines (red), airports (purple), and the San Pedro Bay ports are included.

### 2.2 Model Predictor Variables

The RF models include on-road truck traffic, shipping vessels at the Port of Los Angeles (POLA), airport landings and takeoffs, and rail carloads as indicators of freight activity. Non-truck traffic was included as a non-freight activity predictor. A detailed list of predictor variables and their data sources is available in the supplement (Table S2), and a brief overview is provided here. On-road traffic data was collected from the California Department of Transportation Performance Measurement System (Caltrans PeMS) and aggregated to hourly resolution.^36^ For each site, only the nearest highway was included due to high collinearity of traffic data. Hourly airplane landing and takeoff data was collected from the Federal Aviation Administration’s Traffic Flow Management System Counts database.^37^ Hollywood Burbank Airport (BUR), LAX, Long Beach Airport (LGB), ONT, Palm Springs International Airport (PSP), John Wayne Airport (SNA), and Van Nuys Airport (VNY) were included for all sites.

The number of at-anchor and at-berth vessels at POLA were used to represent freight activity at the San Pedro Bay ports.^38^ Daily carloads from public use sampled waybills for the Los Angeles-Riverside-Orange County, CA-AZ Business Economic Area were used to represent rail activity.^39^ POLA and rail activity data are provided as daily totals. No hourly variability was imposed on the data. Missing data were filled using a 4-day moving average.

While other emission sources (e.g., inland ports, warehouses, and wildfires) are likely to influence measured ozone or NO_*x*_ concentrations at certain locations, these sources are not explicitly represented in the RF models. Their exclusion may contribute to unexplained variability in model performance at some sites. Incorporating additional predictors representing these sources could further improve model skill, discussed in Section 3.1.

Year, day of year, day of week, hour of day, and holiday (yes/no) were included in the model as temporal variables. Site-specific hourly averaged surface pressure, temperature, planetary boundary layer (PBL) height, wind speed and direction, solar radiation at the ground, precipitation, and relative humidity were used as meteorological variables, modeled using the Weather Research and Forecasting Model (WRF v.3.9.1.1) at 4 km resolution.^40^ Meteorological initial and boundary conditions were created using 12km NCEP North American Mesoscale reanalysis model output.^41^ WRF simulations included grid nudging using NCEP ADP Global Observational Weather Data every 3 h, soil nudging, and sea surface temperature updating.^42,43^ A WRF configuration namelist containing physics and dynamics settings is provided in the Supporting Information. WRF evaluation metrics for surface pressure, temperature, wind speed, wind direction, and relative humidity by monitor are available in Table S3. Hourly pressure, temperature, and relative humidity are predicted well overall (*R* ^2^ values of 0.95, 0.89, and 0.75 respectively). Overall wind speed shows moderate predictive accuracy (*R* ^2^ of 0.43) but has a high normalized mean bias (66%). Wind speed mean bias is higher than suggested benchmark statistics from Emery and Tai^44^ but is similar to wind speed bias seen in Los Angeles in Pennington et al.^6^ Wind direction bias is within benchmark statistics. We consider WRF evaluation metrics acceptable.

### 2.3 Model Configuration

Each RF model was trained in MATLAB (r2021a) with the fitrensemble function using a Classification and Regression Trees (CART) algorithm, 600 trees, and bootstrap aggregation. CART evaluates a random subset of predictor variables at each binary node and chooses the predictor that results in the greatest reduction of residual error, continuing until a global minimum residual error is reached or other stop criterion, like maximum number of decision splits, is reached.^45^ Bootstrap aggregation randomly selects samples from the training data set with replacement and trains the decision tree on the bootstrapped sample instead of the original training data set preventing overfitting of training data.^46^ Each model used the “Optimize Hyperparameters” name-value pair to optimize the minimum leaf split, maximum number of decision splits, and the number of predictors to select at random for evaluation at each split. A model configuration sensitivity analysis found that the number of learning cycles and node-splitting algorithm had minimal impact on the root-mean-square error (RMSE) of the resulting model (Figure S1). The input data was split 70% for training and 30% for model testing. Hours with missing predictor data were omitted from both training and testing.

### 2.4 Model Analysis

Model fit was determined by the R^2^ and root mean squared error (RMSE) for the testing data set. Variable importance was assessed through permutation analysis.^47^ This process involved: (1) calculating RMSE on the training data set, (2) randomly shuffling values for one variable while keeping others constant, (3) calculating the resulting RMSE change, and (4) repeating this process 10 times per variable to obtain an average RMSE change which was then normalized by dividing by the initial training set RMSE. Larger average RMSE increases indicated greater variable importance.

Variable impact is considered independent from variable importance. Whereas importance reflects a strong correlative relationship, impact reflects the magnitude of response a variable has on the predicted concentration. To quantify predictor impact, the trained models were used to calculate ozone, NO_*x*_, and O_*x*_ under scenarios in which each freight activity parameter was individually decreased in 10% increments between 10–50%. Scenarios decreasing non-truck traffic provide a non-freight-associated comparison. Additionally, combined freight scenarios at the same 10% increments, in which all freight variables were decreased simultaneously, were run. We quantify the air quality impact of the given sector as the change in concentration between the activity-changed scenario and the actual activity scenario (*C*_model,scenario_ – *C*_model,actual_).

Ozone production regimes were determined using the combined freight activity scenarios during peak ozone hours to represent the impact on ozone under decreasing NO_*x*_ conditions for May to September. Peak ozone hours were defined for each monitor as the hour in which the average hourly ozone concentration reaches a maximum plus 1 h before and after. We assumed ozone changes resulting from decreased freight activity are due to decreases in NO_*x*_ emissions rather than VOC emissions, as the impact of freight VOC emissions is expected to be less significant.^5,12,48,49^ Modeled ozone production regimes were compared with observed ozone production regimes as indicated by weekend-weekday ozone differences, which have previously been used to determine ozone production regimes.^2,3,50^ The observed ozone production regime is determined from the difference between mean hourly weekend (Saturday/Sunday) ozone and weekday (Tuesday/Thursday) observations for each monitor for 2018–2019. The North Hollywood (5) and Long Beach Signal Hill (8) monitors do not have data for 2018–2019 so data from 2021 was used. For both modeled and observed regimes, monitors with average ozone increases greater than 0.5 ppb were considered VOC-limited and monitors with average concentration decreases more than 0.5 ppb were considered NO_*x*_-limited. Monitors with responses between −0.5 and 0.5 ppb were considered transitional/indeterminable. The 0.5 ppb threshold is based on the resolution of ozone observations and the maximum daily 8-h ozone regulatory definition. Both round to the nearest integer mixing ratio, in units of parts per billion.

## 3 Results and Discussion

### 3.1 Model Fit and Variable Importance

Network-aggregated *R* ^2^ values for ozone, NO_*x*_, and O_*x*_ are 0.88, 0.80, and 0.86, respectively, indicating that the models capture most of the temporal variability across the study domain (Figure 2). Aggregated *R* ^2^ is comparable to other LA ML studies for ozone and NO_2_, the closest comparison available.^23,31,51,52^ Note that our *R* ^2^ values are based on hourly concentrations whereas other studies often evaluate daily averaged values. The *R* ^2^ values for individual monitors range from 0.77 to 0.90 for ozone, from 0.56 to 0.82 for NO_*x*_, and from 0.77 to 0.89 for O_*x*_ (Table S4). The North Hollywood (5) and Long Beach Signal Hill (8) monitors had fewer observations than other monitors (both starting operation in 2020), but were included because both included the COVID lockdown period and had *R* ^2^ values comparable to other monitors (Table S4). The normalized mean biases for the NO_*x*_ network and for all individual monitors except Lake Elsinore (monitor 21) are positive and small (Table S4), however the models underestimate high NO_*x*_ concentrations. This underestimation in modeled concentrations suggests that estimated NO_*x*_ impacts (Sections 3.2–3.3) are likely conservative estimates.

**Figure 2:**
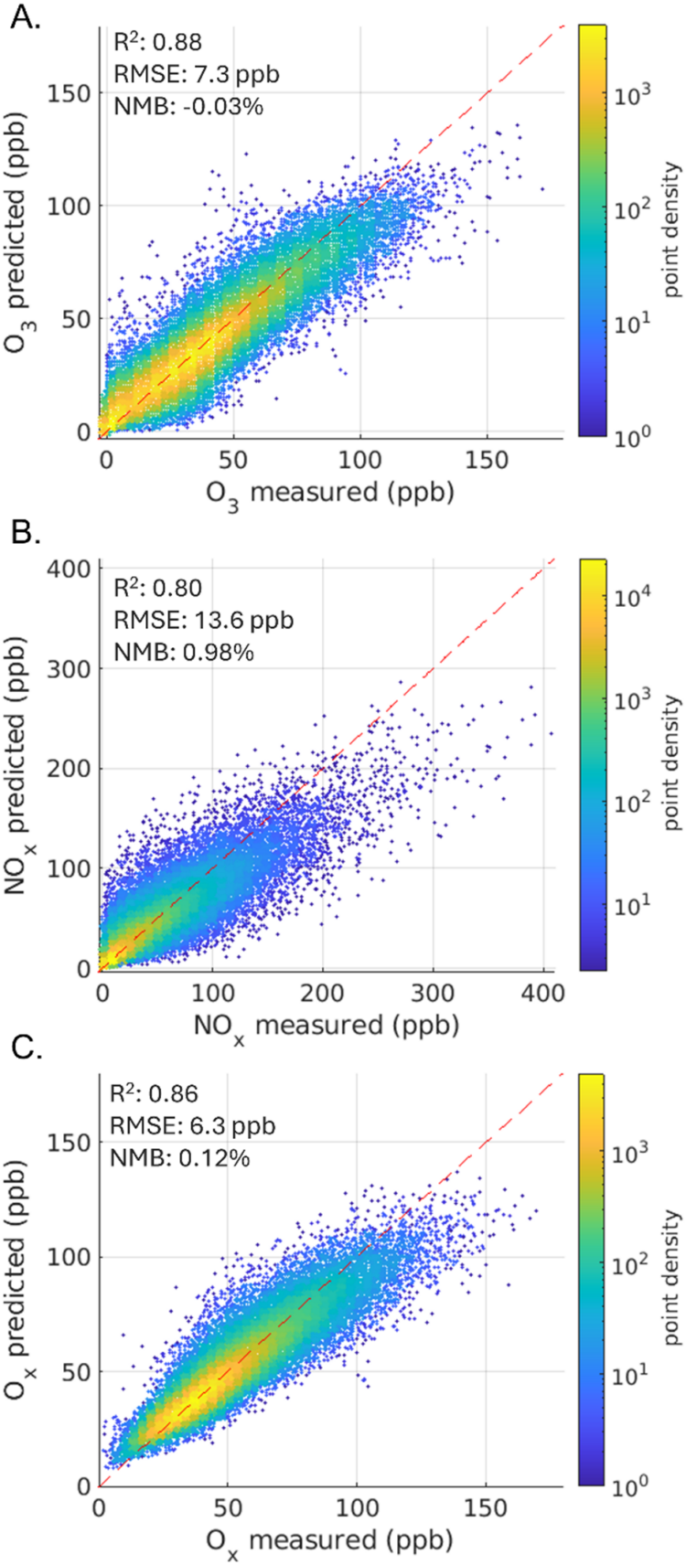
Model fit for hourly test data across all models for (a) ozone, (b) NO_*x*_, and (c) O_*x*_. Model fits are colored by point density. Goodness of fit, root-mean-square error, and normalized mean bias are included.

The most important predictors for ozone are meteorological variables, specifically solar radiation, temperature, and planetary boundary layer (PBL) height (Figure 3). The order of meteorological predictor importance for hourly ozone in this study matches previous studies in Los Angeles.^22,31^ The most important predictors for O_*x*_ are also meteorological variables though the top predictors are temperature, solar radiation, and relative humidity, not PBL height. Important temporal variables for ozone are day of year representing ozone seasonality and hour representing the diurnal trend. Activity variables have lower importance for predicting hourly ozone (Figure S2). Of the activity variables, the POLA, rail, and non-truck traffic are the top predictors, but the average normalized RMSE increase is less than 0.2, less than most of the meteorological predictors. A similar lower importance for emission-related variables is seen by Yang et al.^31^ Agreement of top predictors across studies gives confidence that robust model performance is due to underlying relationships between high importance predictor variables and outcome concentrations as opposed to spurious correlations which may be a concern for low importance predictor variables.

**Figure 3:**
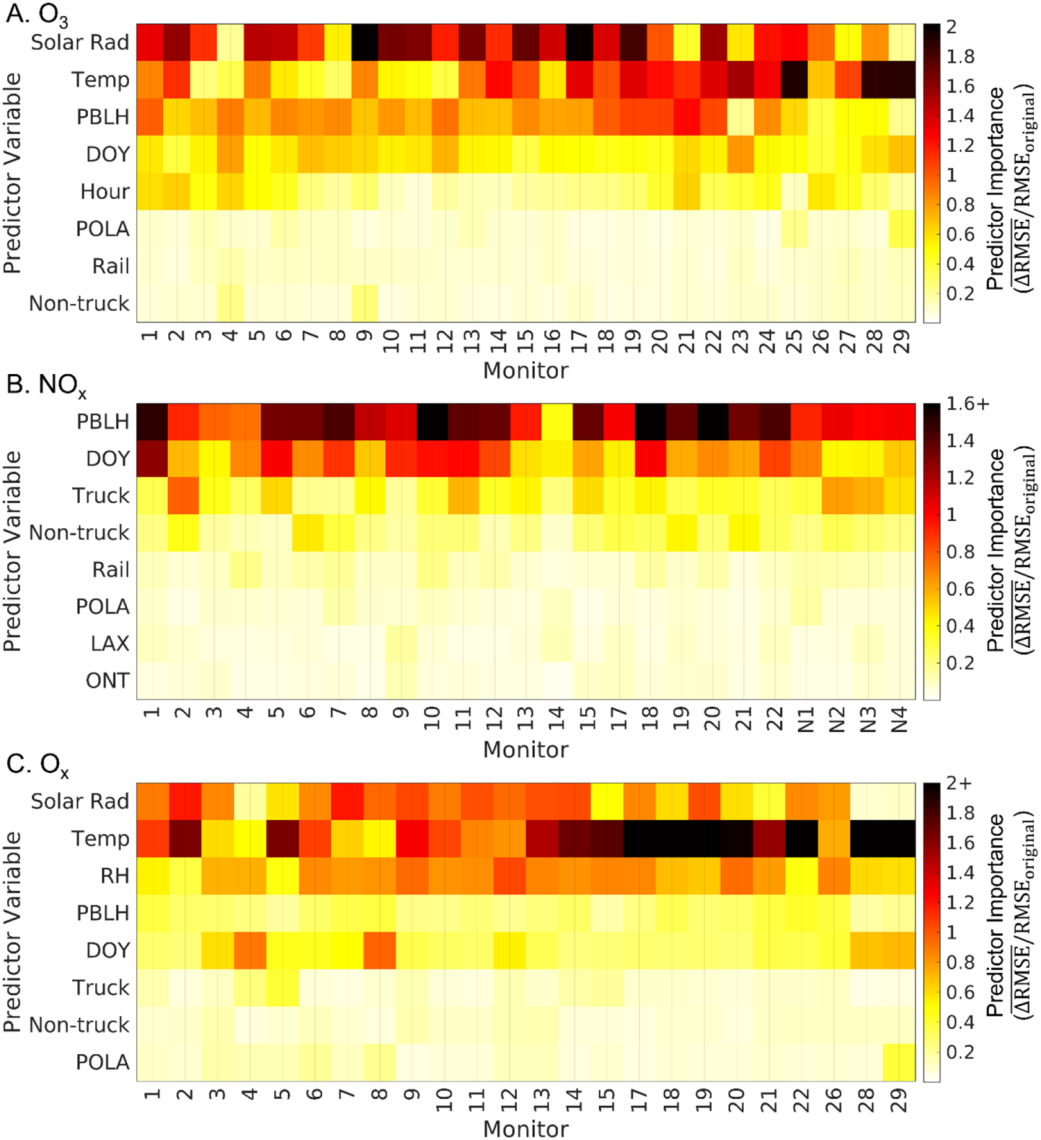
Predictor variable importance for (a) ozone, (b) NO_*x*_, and (c) O_*x*_ for select variables by monitor. Figure S2 shows all predictors.

The most important variables for NO_*x*_ include meteorological, temporal, and activity variables. Averaged across the network, PBL height, day of year, and truck traffic are the top 3 predictors (Figure 3). Truck traffic has a higher importance than non-truck traffic across the network but the LA North Main Street (6), Compton (7), Fontana-Arrow Highway (19), and Lake Elsinore (21) monitors have a higher non-truck traffic importance. Airports have the lowest importance of activity predictors with LAX and ONT leading, however the John Wayne Airport (SNA) importance is notable for the Glendora-Laurel (14) monitor (Figure S2). We consider the high SNA importance for monitor 14 to be the result of a spurious correlation given that the model has a lower *R* ^2^ (0.57) and none of the predictor variables have normalized RMSE increases above 0.5. The lower fit and absence of higher importance values indicate the model is missing predictor variables that better capture NO_*x*_ variability at this location.

### 3.2 RF Models Demonstrate Realistic Diurnal Profiles

The RF models successfully reproduce expected daily patterns in NO_*x*_ and ozone responses to emissions changes, suggesting that machine learning approaches have some ability to reproduce the physical and chemical processes explicitly simulated by CTMs. Modeled diurnal NO_*x*_ concentrations closely follow observations (Figure 4a). We separate the diurnal profiles and the NO_*x*_ concentration responses into two monitor types: near road monitors (red) and all other monitors (black) (Figure 4). The average NO_*x*_ concentration peaks at 41.2 ppb for non-near road monitors and 91.0 ppb for near road monitors. For a 20% freight reduction during the morning peak, average NO_*x*_ reductions range between 1.9 to 4.3 ppb for non-near road monitors and between 5.2 to 8.3 ppb for near road monitors. While the morning concentration peak for both monitor types occur at the same time (0600 to 0800 PT), NO_*x*_ reductions peak slightly earlier for near road sites (0500 to 0600 PT) than for non-near road sites (0600 to 0700 PT). Near road sites exhibit a second peak in reductions during the late afternoon/early nighttime (1600 to 2100 PT) ranging between 3.1 to 5.2 ppb. Low nighttime PBL heights and slower wind speeds lead to NO_*x*_ accumulation in areas near emissions, so it is reasonable that modeled impacts are more pronounced at near-source monitors. During midday hours (1000 to 1500 PT), the concentration response to reduced freight activity is lower across the monitor network likely due to higher PBL height.

**Figure 4:**
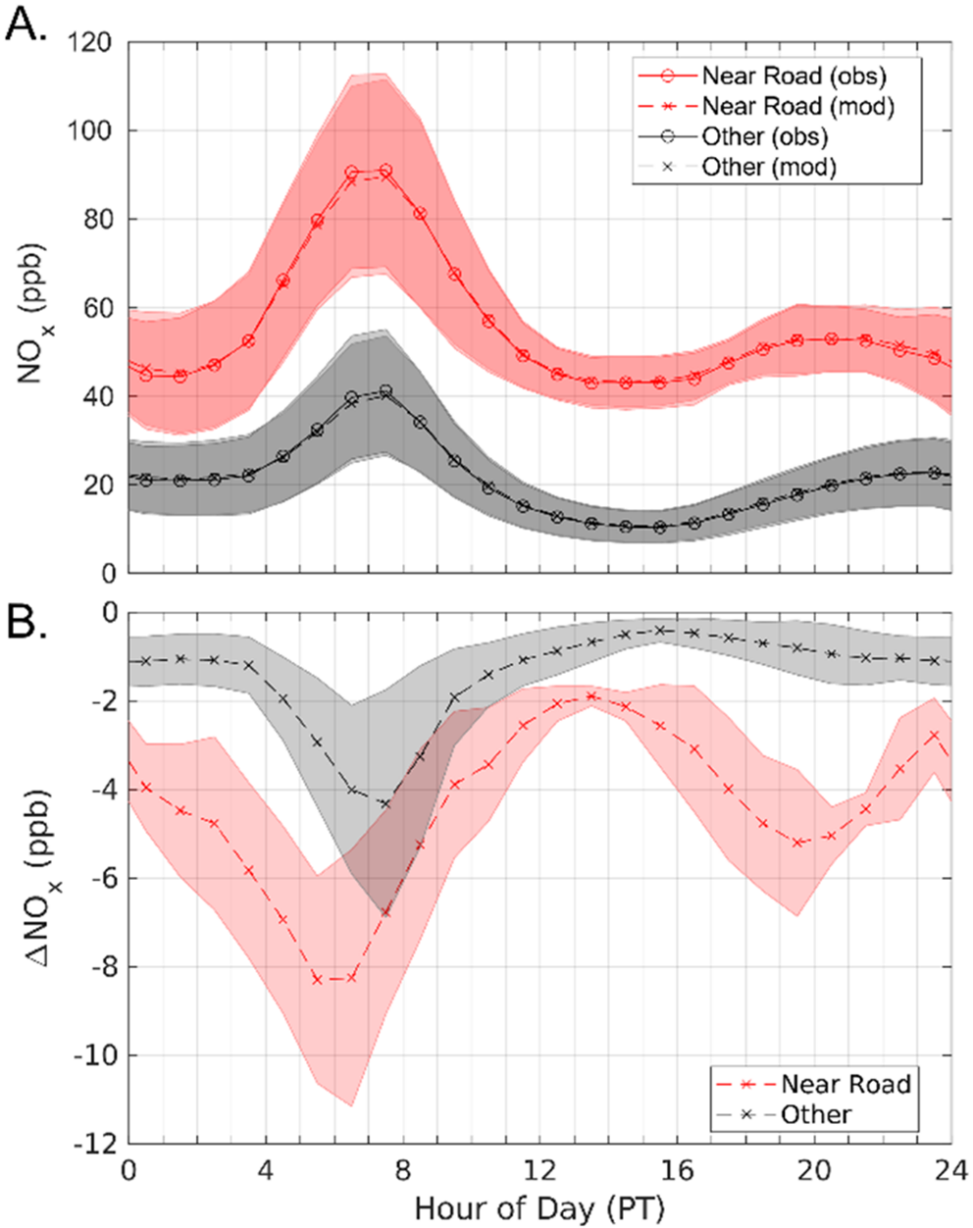
(a) Observed and modeled NO_*x*_ concentration diurnals. (b) NO_*x*_ average concentration changes under 20% freight reduction scenario by time of day. Shaded areas represent the standard deviation of monitor groupings.

Modeled diurnal ozone concentrations are biased high compared to observations for May to September (Figure 5a). This is a seasonal bias not seen for the entire data set (Figures 2 and S5). However, we do not expect that our ozone impacts are biased given that concentration impacts are the difference between the modeled actual and modeled reduced scenarios. From late nighttime to early morning (2200 to 0600 PT), decreases in freight activity result in increased ozone concentrations at all monitor types, reflecting the decreased scavenging of ozone by NO (Figure 5). Starting at 0900 PT, ozone responses differ depending on ozone production regime, with NO_*x*_-limited monitors showing decreases in ozone and VOC-limited monitors showing increases. Transitional monitors show initial ozone increases from 0900 to 1100 PT, but by 1300 PT show nearly no change in ozone. Note that although ozone increases under VOC-limited conditions, daytime O_*x*_, which is related to downwind ozone production capability, decreases under reduced freight conditions for all ozone regimes indicating lowering freight emissions lowers ozone concentration on a regional scale (Figure S4). Section 3.4 further discusses midday ozone concentration impacts from reduced freight activity.

**Figure 5:**
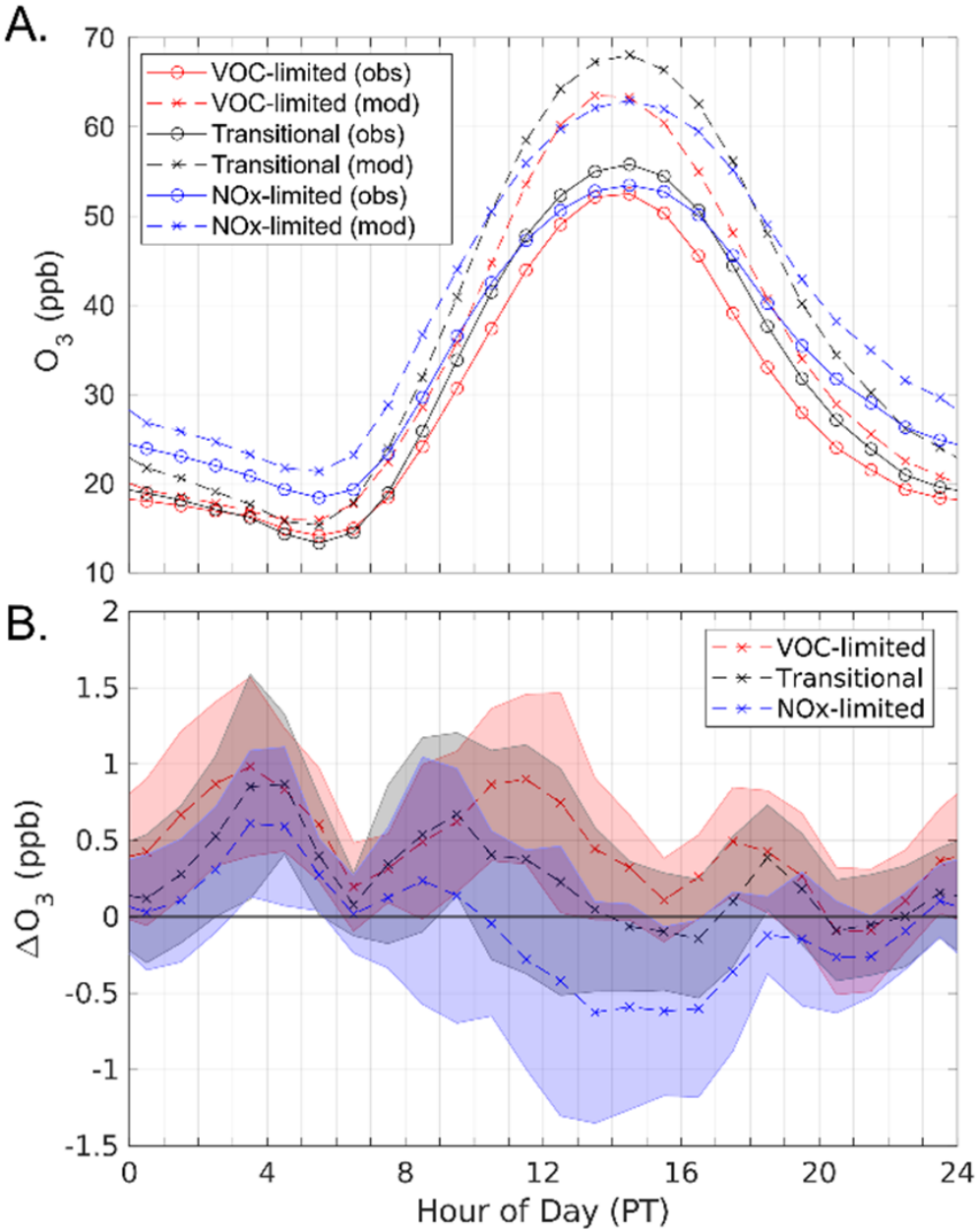
(a) Observed and modeled ozone concentration diurnals for May to September. (b) Ozone average concentration changes under 20% freight reduction conditions for the same time period. Monitors are split into VOC-limited (red), NO_*x*_-limited (blue), and transitional (black) production regimes. Shaded areas represent the standard deviation of monitor groupings. Due to large modeled and observed ozone standard deviations, standard deviations are omitted from panel a but are available in supplementary (Figure S3).

### 3.3 Freight-Specific NO_*x*_ Concentration Impacts

Figure 6 shows the impact of individual sectors on NO_*x*_ concentrations at each monitor, and the relative importance of freight versus non-truck traffic. During morning hours (0600–0800 PT), when concentration responses to emissions are pronounced for all monitors, the relative impact of freight and non-truck sectors on NO_*x*_ was similar under different activity reduction magnitudes (Figure S6). The freight contribution estimates (Figure 6b) are averaged across multiple reduction scenarios (10 to 50%).

**Figure 6:**
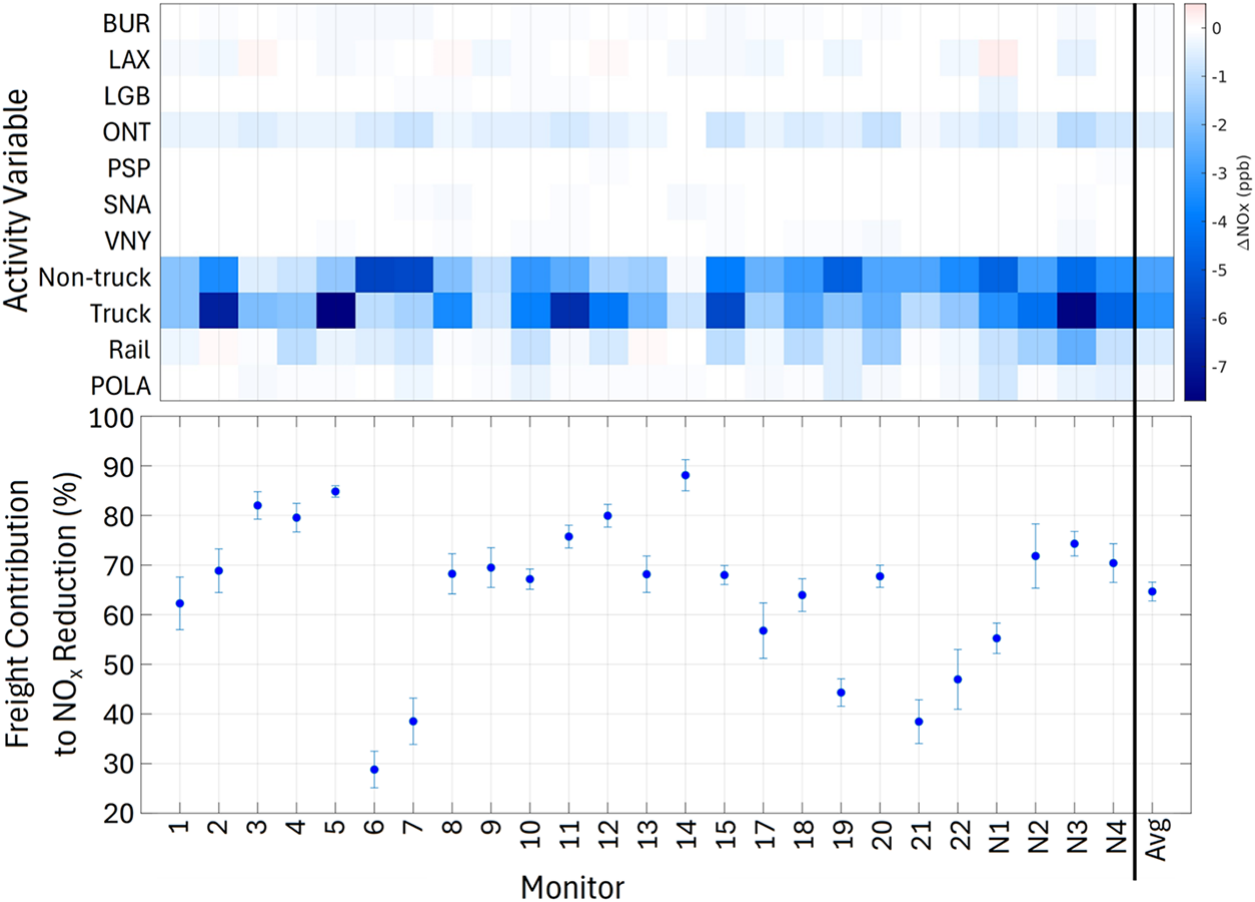
(a) NO_*x*_ concentration impacts by activity variable for 6–8 am PT under 20% decreased activity scenarios. (b) Freight contribution to total NO_*x*_ reductions. Error bars are standard deviations of modeled estimates.

It is important to note that a variable’s importance ranking (Figure 3) does not necessarily correspond to the magnitude of response it creates when perturbed (Figure 6). For example, decreasing ONT has a greater NO_*x*_ reduction than decreasing LAX or POLA for most monitors despite a lower permutation importance for the ONT predictor variable. The difference between importance and impact results from the use of binary splits in RF model predictions. Within each decision tree, predictor variables are used to make a series of binary splits that lead to the prediction of the outcome variable. The average of all predictions in a forest is the final prediction. Because the split is binary at each node, variables can be preferentially chosen at multiple nodes due to larger decreases in residual error (high importance), but cause small differences in the final prediction when averaged across the forest (low impact). Thus, while model importance indicates which variables are most useful for making accurate predictions, perturbation scenarios are better for estimating changes in the outcome variable when predictors are modified.

Truck and non-truck traffic have the largest impact on NO_*x*_ concentrations with nontrivial impacts from rail and the ONT airport. The LA North Main Street (6), Compton (7), and Lake Elsinore (21) monitors have larger non-truck traffic NO_*x*_ impacts than total freight impacts. The network average freight contribution (65% ± 2%) suggests that freight sources contribute more to NO_*x*_ concentrations compared to non-truck traffic in the LA basin. We consider this freight contribution an upper estimate of annual totals as models omit weekend observations when freight emissions are lower.

Most of the network wide freight contribution is driven by truck and rail activity. The models suggest ONT activity accounts for ∼10% of the total freight impact, with nearly every monitor showing sensitivity to ONT activity. This nonphysical result could be caused by correlations between flight activity at ONT and regional freight activity. While passenger volumes decreased in 2020 due to COVID, the number of flights at ONT quickly recovered within a few months, leading to minimal change in the year’s diurnal profile compared to previous years (Figure S7). ONT flight recovery was likely due to increased freight tonnage transported considering ONT freight tonnage peaked in 2020 and continued to be higher than pre-COVID averages in 2021.^53^ This example illustrates the need for careful interpretations of ML model inputs and results.

The Port of Los Angeles has little network wide impact on NO_*x*_ concentrations (max of 4% of total freight impact averaged across network), but the largest POLA impacts for every reduction scenario occurred at the Long Beach Route 710 (N1) monitor, the near-road monitor closest to the port. POLA impacts are ∼11% of the freight impact at monitor N1 (representing 4–7% of total modeled NO_*x*_ impact at this site). This result matches a previous measurement study by Mousavi et al.^54^ that suggested emissions from ports impact nearby communities but port impacts for the larger LA basin are small relative to other sources.

Our results align with the 2020 CARB mobile source strategy regarding the importance of considering NO_*x*_ emission reduction strategies that include freight emissions, especially for truck and rail sources.^11^ Current California vehicle electrification goals phase out the sale of internal combustion passenger vehicles by 2035.^55^ Our results suggest that NO_*x*_ emissions from passenger vehicle electrification will have the highest impact in areas with the highest population density where non-truck traffic emissions dominate (e.g., downtown Los Angeles), but for basin-wide NO_*x*_ reductions, truck and rail emissions may have a higher impact for the same relative reduction.

### 3.4 Modeled Ozone Production Regimes

Reduced freight model scenarios suggest that during peak ozone hours, coastal monitors (3 and 8) are VOC-limited or transitional (monitor 4) (Figure 7). Monitors north of Los Angeles (1, 2, and 5) are transitional. For the monitors between Los Angeles and San Bernardino that run along I-10 and I-210, the monitors closer to Los Angeles (13–15), are VOC-limited whereas the monitors further east (17, 18, and 20) are transitional. Similarly, the Orange County monitor closest to downtown LA (11) is VOC-limited but the monitors further south (12 and 16) are transitional. The monitors in San Bernardino (22, 23, and 25) show different ozone production regimes. Monitors south and east of San Bernardino (21, 24, 26, 27, and 29) are NO_*x*_-limited or transitional (monitor 28).

**Figure 7:**
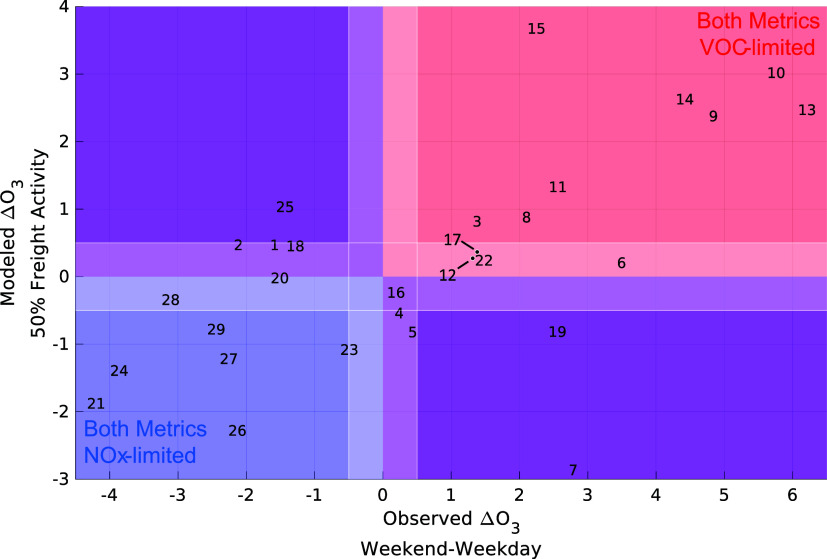
Ozone concentration changes (May to September) during peak ozone hours for 50% combined freight activity scenario vs weekday/weekend effect calculated from observations.

In general, observation-based weekday/weekend ozone production regime assessments align with regime assessments from freight perturbation analysis. Three of the 29 sites have opposite modeled ozone production regimes compared to the weekday/weekend assessment. These differing results could reflect a lower impact of freight transit at the monitor location. For example, weekday/weekend results suggest the LA North Main Street (6) and Compton (7) monitors are in VOC-limited locations, whereas freight perturbation models suggest the Main Street site is transitional and the Compton site is NO_*x*_-limited. However, the overall impact of freight on NO_*x*_ at these locations is smaller than other sites (Figure 6b). If, instead, non-truck NO_*x*_ is perturbed, these locations display VOC-limited responses (Figure S8) in agreement with the weekday/weekend trends making the monitors in south and east LA (6–7 and 9–10) all VOC-limited. The Lake Elsinore (21) and Redlands (25) monitors also agree with the weekday/weekend trends in the non-truck reduction scenario. Note that the Redland monitor does not measure NO_*x*_ so it was not evaluated for freight contribution to NO_*x*_. The Fontana-Arrow Highway monitor (19) is the only monitor that remains misaligned compared to the weekday/weekend assessment as it is transitional in the non-truck reduction scenario. We consider the overall agreement between the weekday/weekend assessment and the freight perturbation analysis to support the capability of ML modeled ozone production regimes.

Our models suggest that Los Angeles’ urban core and nearby downwind areas are still VOC-limited during peak ozone hours. For two San Bernardino monitors (22 and 23), the ozone production regime changes depending on time of day. The downtown San Bernardino monitor (22) starts VOC-limited and becomes transitional in later hours and the Crestline monitor (23) is VOC-limited during the morning and early afternoon (10–13 PT) and becomes NO_*x*_-limited during later hours (14–17 PT). We hypothesize it is due to higher NO_*x*_ concentrations during early and midday hours because of a combination of nearby emissions and wind-advected concentrations but by later hours NO_*x*_ concentrations fall enough to cause a regime transition.^5^

The freight perturbation analysis does not predict future changes in ozone production regime as more extreme reductions did not result in regime changes during peak ozone hours from VOC-limited to transitional or NO_*x*_-limited for any sites. Do et al.^23^ came to a similar conclusion on the unreliability of ozone RF models to predict future scenarios under new regimes meaning that unless the COVID reductions caused a regime change, it is unlikely that our model would be able to show such theoretical future changes. Previous Pasadena model sensitivities suggest that although leaving a VOC-limited regime could start at ∼30% NO_*x*_ emissions reductions, ozone concentrations will not start to decrease relative to the present until at least 70% NO_*x*_ emissions reductions supporting our conclusion.^56^ However, unlike other methods that are limited by multiyear lags behind present day observations,^3^ we anticipate that RF models are capable of adjustment as more observations become available under transitional regimes allowing the potential to assess ozone regime changes closer to real time.

## 4 Conclusion

Here we show the capability of random forest (RF) models to determine freight specific impacts on NO_*x*_ concentrations and to determine ozone production regimes in Los Angeles (LA). We also show that RF models can reproduce expected daily patterns in NO_*x*_ and ozone responses to emissions changes. This points to an ability of machine learning (ML) methods to provide an accurate assessment of the sensitivity of observed concentrations to specific activity-related emission sources. Future research should further compare predictor-outcome relationships from ML models to corresponding emissions-concentration relationships from traditional chemical transport models.

Of the activity predictor variables, we find that truck and non-truck traffic have the largest impact on NO_*x*_ concentrations in LA. While lower freight impacts were observed at select monitors, LA North Main Street (6), Compton (7), and Lake Elsinore (21), network freight impacts emphasize the importance of NO_*x*_ emission reduction strategies that include freight emissions. Activity reduced model scenarios suggest that during peak ozone hours, the LA urban core and nearby downwind areas remain VOC-limited in 2021. Though ozone regime transitions were not modeled for VOC-limited areas, we anticipate that RF models are capable of near-real time adjustment as more observations become available and NO_*x*_ emissions continue to decrease.

ML studies require many observations and sufficient variability in the training data set. Areas like Los Angeles that have a network of ground monitors with high temporal resolution and historical data coverage are ideal for machine learning analyses and disruptions due to COVID-19 lockdown provided predictor variability. In less well-monitored areas, future research could leverage the growing number of satellite-derived observations and low-cost censors alongside other abnormal events. This study supports the growing potential of machine learning for scientific and policy relevant applications.

## Supplementary Material





## Notes

California Air Resources Board ground monitor information (Table S1); sensitivity analysis to number of trees and node-splitting algorithm on sample monitor (Figure S1); predictor variable information (Table S2); WRF evaluation metrics (Table S3); *R* ^2^, RMSE, and NMB for all models (Table S4); predictor importance across all monitors for all variables (Figure S2); observed and modeled ozone concentration diurnals for May to September with standard deviations of monitor groupings (Figure S3); observed and modeled O_*x*_ average concentration diurnals and concentration changes under 20% freight reduction conditions (Figure S4); observed and modeled ozone and O_*x*_ for entire period (Figure S5); NO_*x*_ concentration impacts by activity variable for 6–8 am PT under additional decreased activity scenarios (Figure S6); Airport landing/takeoff diurnals for LAX, ONT, and LGB (Figure S7); Ozone regime analysis using non-truck traffic reductions for select monitors (Figure S8) (PDF)
WRF input namelist (TXT)

## §

U.S. Environmental Protection Agency, Research Triangle Park, North Carolina 27709, United States
