# Supplementary material for: Comparative Impacts of Freight and Non-truck Traffic on NO x and Ozone Concentrations in the Los Angeles Basin
Source: ACS EST Air. 2026 Jan 21;3(2):548–57. doi: 10.1021/acsestair.5c00396 (PMC12910605; doi:10.1021/acsestair.5c00396)
Supplement: Supplementary file 1 [file ea5c00396_si_001.pdf]

Supporting Information for

Comparative Impacts of Freight and Non-truck Traffic on NO_x and Ozone Concentrations in the Los Angeles Basin

Aryiana C. Moore[1], T. Nash Skipper[1,2], Armistead G. Russell[1], Jennifer Kaiser[1,3]*

1. School of Civil and Environmental Engineering, Georgia Institute of Technology, Atlanta, Georgia 30332, United States

2. Now at: U.S. Environmental Protection Agency, Research Triangle Park, NC 27709, USA

3. School of Earth and Atmospheric Sciences, Georgia Institute of Technology, Atlanta, Georgia 30332, United States

* Email: jennifer.kaiser@ce.gatech.edu

#	AQS ID	Monitor Name	Lat	Lon	# of Observations		
					O_3	NO_x	O_x
1	060371201	Reseda	34.20	-118.53	23,927	24,150	23,906
2	060376012	Santa Clarita	34.38	-118.53	24,103	24,067	23,988
3	060370113	West Los Angeles VA Hospital	34.05	-118.46	24,178	22,083	21,914
4	060375005	Los Angeles: Westchester Parkway	33.96	-118.43	22,273	21,913	21,722
5	060374010	North Hollywood	34.18	-118.36	12,090	12,099	12,020
6	060371103	Los Angeles: North Main Street	34.07	-118.23	23,819	23,902	23,552
7	060371302	Compton	33.90	-118.20	23,941	23,612	23,344
8	060374009	Long Beach: Signal Hill	33.79	-118.17	11,732	11,996	11,695
9	060372005	Pasadena	34.13	-118.13	24,233	24,038	23,949
10	060371602	Pico Rivera	34.01	-118.07	23,977	24,117	23,922
11	060595001	La Habra	33.93	-117.95	23,969	23,730	23,665
12	060590007	Anaheim: Pampas Lane	33.83	-117.94	24,449	24,293	24,288
13	060370002	Azusa	34.14	-117.92	24,045	23,887	23,769
14	060370016	Glendora-Laurel	34.14	-117.85	23,960	23,803	23,466
15	060371701	Pomona	34.07	-117.75	24,220	24,201	24,097
16	060592022	Mission Viejo	33.63	-117.68	24,417		
17	060711004	Upland	34.10	-117.63	23,805	23,290	23,226
18	060658005	Mira Loma Van Buren	34.00	-117.49	23,999	23,581	23,503
19	060712002	Fontana-Arrow Highway	34.10	-117.49	23,995	23,978	23,849
20	060658001	Riverside-Rubidoux	34.00	-117.42	23,849	23,699	23,678
21	060659001	Lake Elsinore	33.68	-117.33	24,154	23,721	23,677
22	060719004	San Bernardino	34.11	-117.27	24,132	24,156	24,072
23	060710005	Crestline	34.24	-117.27	24,358		
24	060656001	Perris	33.79	-117.23	23,625		
25	060714003	Redlands-Dearborn	34.06	-117.15	24,436		
26	060650009	Temecula-Techanga Tribe	33.45	-117.09	21,247		17,236
27	060650016	Winchester	33.58	-117.07	24,227		
28	060650012	Banning Airport	33.92	-116.86	24,154		23,743
29	060651016	AMS-Morongo Boneyard	33.95	-116.83	23,880		20,502
N1	060374008	Long Beach: Route 710	33.86	-118.20		23,733	
N2	060590008	Anaheim: 812 W Vermont Street	33.82	-117.92		23,578	
N3	060710027	Ontario: Route 60	34.03	-117.62		23,669	
N4	060710026	Ontario: NW Corner I-10 and Etiwanda Ave	34.07	-117.53		23,022	

Table S1. California Air Resources Board ground monitor information

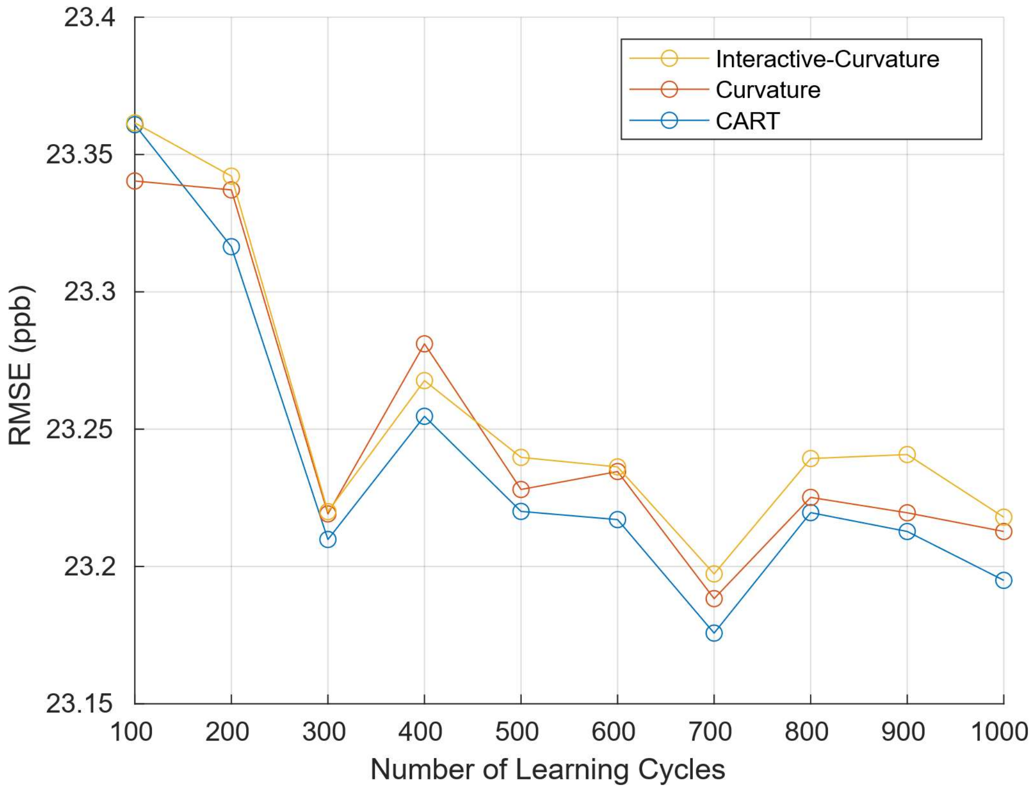

Figure S1. Sensitivity analysis to number of learning cycles and node-splitting algorithm on sample monitor. Note that the range of RMSE is less than 0.5 ppb.

Predictor Variable	Unit	Temporal Resolution	Min	Max	Data Source
Activity Variables					
Truck (only nearest highway selected for model)					
Interstate 5	Vehicle Miles Traveled	hourly	0	75867	California Department of Transportation Performance Measurement System (https://pems.dot.ca.gov/)
Interstate 10	Vehicle Miles Traveled	hourly	0	89898	
Interstate 15	Vehicle Miles Traveled	hourly	0	43883	
State Route 60	Vehicle Miles Traveled	hourly	0	47107	
Interstate 110	Vehicle Miles Traveled	hourly	0	19969	
Interstate 210	Vehicle Miles Traveled	hourly	0	64549	
Interstate 215	Vehicle Miles Traveled	hourly	0	23983	
Interstate 405	Vehicle Miles Traveled	hourly	0	53889	
Interstate 710	Vehicle Miles Traveled	hourly	0	25128	
Nontruck (only nearest highway selected for model)					
Interstate 5	Vehicle Miles Traveled	hourly	0	1425333	California Department of Transportation Performance Measurement System (https://pems.dot.ca.gov/)
Interstate 10	Vehicle Miles Traveled	hourly	0	2085956	
Interstate 15	Vehicle Miles Traveled	hourly	0	808459	
State Route 60	Vehicle Miles Traveled	hourly	0	890736	
Interstate 110	Vehicle Miles Traveled	hourly	0	398628	
Interstate 210	Vehicle Miles Traveled	hourly	0	949270	
Interstate 215	Vehicle Miles Traveled	hourly	0	538014	
Interstate 405	Vehicle Miles Traveled	hourly	0	1172914	
Interstate 710	Vehicle Miles Traveled	hourly	0	258572	
Hollywood Burbank Airport (BUR)	Airplane Landings/Takeoffs	hourly	0	118	Federal Aviation Administration Traffic Flow Management System Counts (https://www.aspm.faa.gov/tfms/sys/OPSNET.asp)
Los Angeles International Airport (LAX)	Airplane Landings/Takeoffs	hourly	0	189	
Long Beach Airport (LGB)	Airplane Landings/Takeoffs	hourly	0	327	
Ontario International Airport (ONT)	Airplane Landings/Takeoffs	hourly	0	97	
Palm Springs International Airport (PSP)	Airplane Landings/Takeoffs	hourly	0	64	
John Wayne Airport (SNA)	Airplane Landings/Takeoffs	hourly	0	188	
Van Nuys Airport (VNY)	Airplane Landings/Takeoffs	hourly	0	166	
Port of Los Angeles (POLA)	Vessels at Berth or Anchor	daily (weekdays only)	0	59	Port of Los Angeles Historical Container Vessel Activity (https://www.portoflosangeles.org/business/supply-chain/ships)
Rail	Estimated Carloads	daily	590	28913	Surface Transportation Board 2023 Public Use Waybille Sample (https://www.stb.gov/reports-data/waybill/)
Meteorologic Variables					
Surface Pressure	Pa	hourly	84733.0	102673.7	Weather Research and Forecasting Model (WRFv.3.9.1.1)
Temperature (at 2 m)	K	hourly	268.6	320.7	
Planetary Boundary Layer Height	m	hourly	28.5	5466.5	
Wind Speed (at 10 m)	m/s	hourly	0.0	17.9	
Wind Direction (at 10 m)	degree	hourly	0.0	360.0	
Solar Radiation (that reaches surface)	W/m^2	hourly	0.0	1126.6	
Precipitation	cm	hourly	0	2.99	combined nonconvective and convective precipitation from WRF v.3.9.1.1
Relative Humidity	%	hourly	2.3	105.5	calculated using water ratio at 2 m, surface pressure, and temperature at 2 m data from WRF v.3.9.1.1
Temporal Variables					
Year	[-]	annual	2018	2021	N/A
Day of Year	[-]	daily	1	366	N/A
Day of Week	[-]	daily	2	6	N/A
Hour of Day	[-]	hourly	0	23	N/A
Holiday	[-]	daily	0	1	N/A

Table S2. Predictor variable information

#	Monitor Name	Pressure		Temperature		Wind Speed			Wind Direction		Relative Humidity	
		R^2	NMB (%)	R^2	NMB (%)	R^2	NMB (%)	MB (m/s)	NMB (%)	MB (deg)	R^2	NMB (%)
1	Reseda			0.88	5.8	0.37	127	1.2	11	20	0.74	-17
2	Santa Clarita	0.93	-0.88	0.87	5.6	0.36	58	1.2	27	45	0.74	-16
3	West Los Angeles VA Hospital	0.94	-0.37	0.79	5.7	0.32	145	1.4	5.9	13	0.63	-15
5	North Hollywood			0.87	5.8	0.44	72	0.92	13	24	0.71	-21
6	Los Angeles: North Main Street	0.71	-0.30	0.85	5.6	0.45	4	0.07	13	22	0.69	-9.2
7	Compton	0.87	-0.03	0.82	5.7	0.58	31	0.45	4.9	10	0.67	-11
8	Long Beach: Signal Hill	0.37	-0.24	0.83	5.5	0.54	26	0.43	7.7	16	0.67	-7.3
9	Pasadena			0.85	5.7	0.29	198	1.4	11	21	0.74	-16
10	Pico Rivera	0.79	-0.21	0.87	5.7	0.39	27	0.46	21	32	0.68	-16
11	La Habra			0.86	5.5	0.45	74	0.93	0.45	0.86	0.71	-13
12	Anaheim: Pampas Lane	0.65	-0.06	0.85	5.6	0.54	30	0.46	3.0	5.8	0.70	-11
13	Azusa	0.88	0.21	0.88	5.6	0.33	53	0.76	1.7	3.2	0.71	-8.8
14	Glendora-Laurel			0.90	5.5	0.33	172	1.6	32	44	0.76	-11
15	Pomona					0.52	97	1.2	12	24		
16	Mission Viejo	0.85	-0.19	0.87	5.3	0.34	87	1.1	5.7	11	0.70	-5.4
17	Upland	0.83	0.09	0.91	5.6	0.43	74	1.2	4.9	11	0.77	-20
18	Mira Loma Van Buren	0.87	-0.15	0.91	5.6	0.60	77	1.3	8.2	17	0.79	-14
19	Fontana-Arrow Highway	0.88	0.03	0.91	5.7	0.60	48	0.95	8.0	15	0.78	-18
20	Riverside-Rubidoux	0.75	-0.29	0.91	5.5	0.52	93	1.3	1.6	3.5	0.80	-12
21	Lake Elsinore			0.93	5.6	0.33	127	1.2	8.2	18	0.78	-14
22	San Bernardino			0.91	5.6	0.50	52	0.75	13	23	0.78	-17
23	Crestline			0.87	5.7	0.15	245	3.4	-2.5	-5.0	0.68	-15
24	Perris	0.73	0.07	0.92	5.4	0.37	57	0.76	11	23	0.82	-6.3
25	Redlands-Dearborn			0.92	5.8	0.34	103	1.3	7.3	14	0.79	-17
27	Winchester			0.92	5.4	0.43	14	0.35	8.3	15	0.80	-12
28	Banning Airport	0.95	-0.90	0.94	5.3	0.50	34	1.4	-5.5	-13	0.79	-13
N1	Long Beach: Route 710 Near Road			0.82	5.5	0.53	23	0.37	6.4	13	0.68	-6.3
N2	Anaheim: 812 W Vermont Street			0.87	5.3	0.55	39	0.56	9.7	17.3	0.73	-5.3
N3	Ontario: Route 60 Near Road	0.89	-0.01	0.91	5.4	0.52	110	1.3	20	40	0.79	-12
N4	Ontario: NW Corner I-10 and Etiwanda Ave	0.94	0.09	0.91	5.6	0.59	27	0.57	17	30	0.78	-12
Overall		0.98	-0.18	0.89	5.6	0.43	66	1.02	8.5	16	0.75	-12

Table S3. WRF evaluation for pressure, temperature, wind speed and direction, and relative humidity. Ground observations for monitors were collected from the Air Quality System Data Mart. Goodness of fit is omitted for wind direction because wind direction is in degrees which is not linear. Mean bias is included for wind speed and direction for ease of comparison with other studies.

#	Monitor Name	R^2			RMSE (ppb)			NMB (%)		
		O_3	NO_x	O_x	O_3	NO_x	O_x	O_3	NO_x	O_x
1	Reseda	0.86	0.78	0.83	7.9	9.9	6.6	-0.3	1.6	-0.2
2	Santa Clarita	0.88	0.72	0.87	7.7	6.1	7.1	-0.4	1.7	0.1
3	West Los Angeles VA Hospital	0.79	0.63	0.78	7.1	9.1	5.4	0.0	2.2	0.6
4	Los Angeles: Westchester Parkway	0.78	0.69	0.77	6.6	9.8	4.6	-0.4	1.4	0.2
5	North Hollywood	0.87	0.82	0.85	7.6	10.7	6.3	-0.3	0.7	0.8
6	Los Angeles: North Main Street	0.86	0.78	0.82	6.8	14.0	5.7	0.1	0.4	0.3
7	Compton	0.82	0.80	0.78	6.8	17.5	5.4	0.6	2.2	0.1
8	Long Beach: Signal Hill	0.77	0.75	0.78	7.2	11.7	5.2	0.0	2.3	0.6
9	Pasadena	0.87	0.74	0.86	7.3	8.3	6.7	-0.5	1.9	0.2
10	Pico Rivera	0.86	0.79	0.84	7.1	13.5	6.0	-0.1	2.2	0.1
11	La Habra	0.85	0.80	0.83	7.2	10.9	6.2	-0.5	0.8	-0.2
12	Anaheim: Pampas Lane	0.83	0.77	0.79	6.9	12.3	5.4	-0.1	2.0	-0.2
13	Azusa	0.85	0.56	0.87	7.7	10.9	6.5	-0.5	1.1	0.2
14	Glendora-Laurel	0.87	0.57	0.89	7.3	6.1	7.0	-0.1	1.1	0.02
15	Pomona	0.90	0.77	0.86	6.7	12.9	6.3	0.5	0.9	0.2
16	Mission Viejo	0.79			6.7			0.3		
17	Upland	0.89	0.71	0.88	7.6	8.2	6.7	0.1	0.9	0.1
18	Mira Loma Van Buren	0.89	0.82	0.88	7.7	12.1	6.6	-0.1	1.7	0.1
19	Fontana-Arrow Highway	0.91	0.74	0.88	7.4	12.2	6.8	0.3	1.4	-0.1
20	Riverside-Rubidoux	0.90	0.80	0.88	7.3	14.2	6.1	-0.1	1.9	0.4
21	Lake Elsinore	0.87	0.79	0.84	7.3	5.4	6.3	0.5	-3.1	0.01
22	San Bernardino	0.91	0.78	0.89	8.0	9.1	7.1	-0.1	0.04	0.15
23	Crestline	0.84			8.9			0.2		
24	Perris	0.89			7.7			-0.1		
25	Redlands-Dearborn	0.90			7.6			0.4		
26	Temecula-Techanga Tribe	0.85		0.84	6.4		6.3	0.4		0.2
27	Winchester	0.82			5.7			-0.2		
28	Banning Airport	0.83		0.81	8.2		6.9	-0.1		-0.3
29	AMS-Morongo Boneyard	0.81		0.82	6.8		6.5	-0.2		0.3
N1	Long Beach: Route 710 Near Road		0.73			29.4			<0.01	
N2	Anaheim: 812 W Vermont Street		0.69			13.5			0.5	
N3	Ontario: Route 60 Near Road		0.69			22.4			0.2	
N4	Ontario: NW Corner I-10 and Etiwanda Ave		0.69			21.1			0.8	

Table S4. Goodness of fit (R^2), root mean square error (RMSE), and normalized mean bias (NMB) for all models

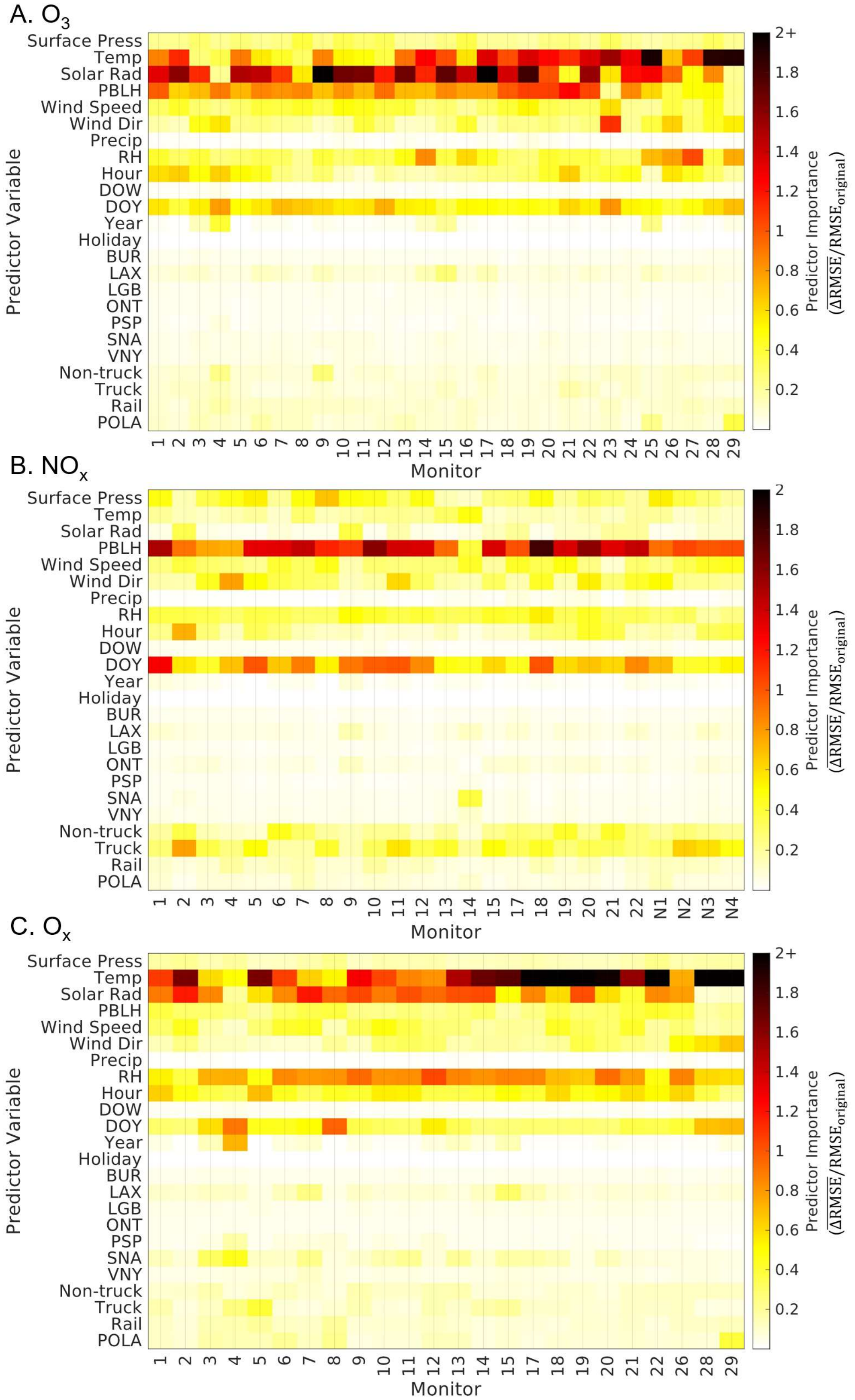

Figure S2. Predictor variable importance across all monitors for all variables for a) ozone, b) NO_x, and c) O_x.

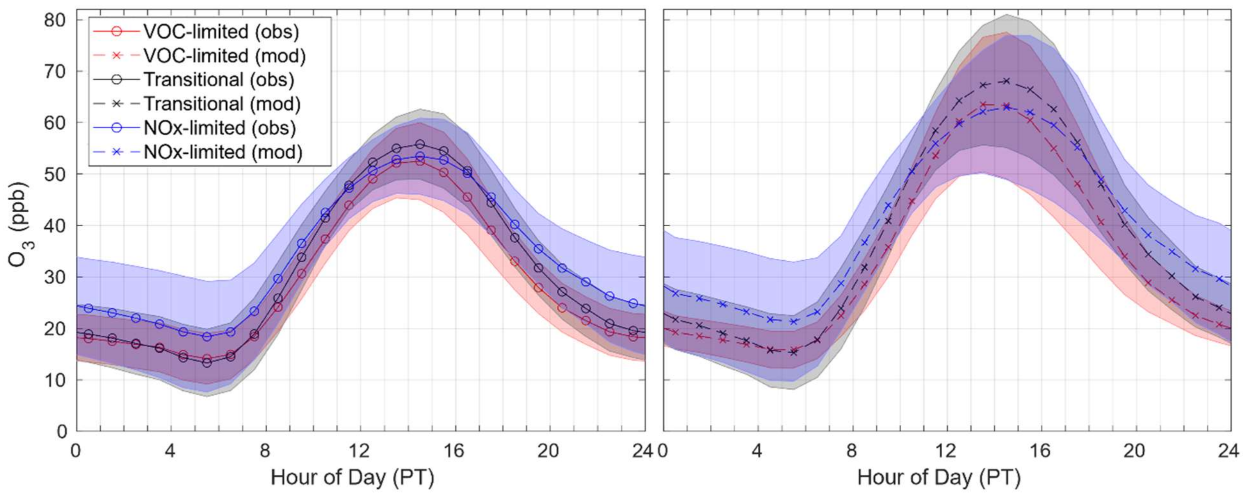

Figure S3. Observed (left) and modeled (right) ozone concentration diurnals for May to September. Monitors are split into VOC-limited (red), NO_x-limited (blue), and transitional (black) ozone production regimes. Shaded areas represent the standard deviation of monitor groupings.

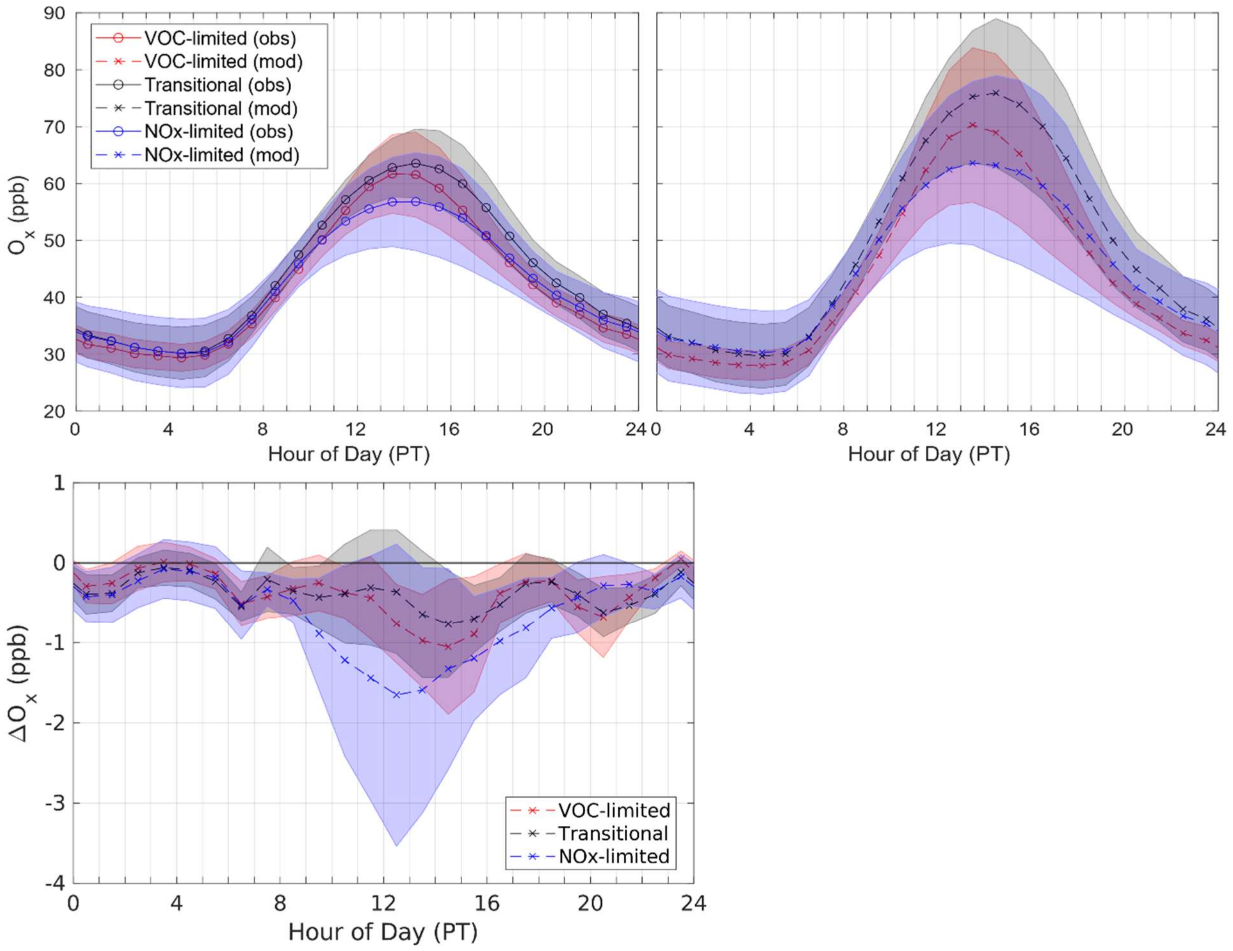

Figure S4. O_x (NO_2+ozone) average concentration changes for May to September under 20% freight reduction conditions. Monitors are split into VOC-limited (red), NO_x-limited (blue), and transitional (black) ozone production regimes. Shaded areas represent the standard deviation of monitor groupings.

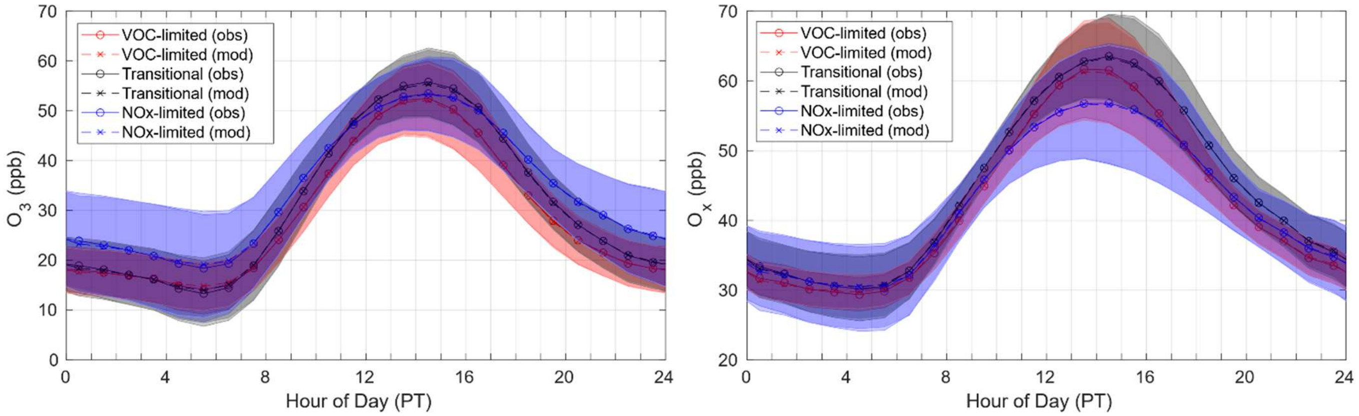

Figure S5. Observed and modeled ozone (left) and Ox (right) concentration diurnals for the entire period.

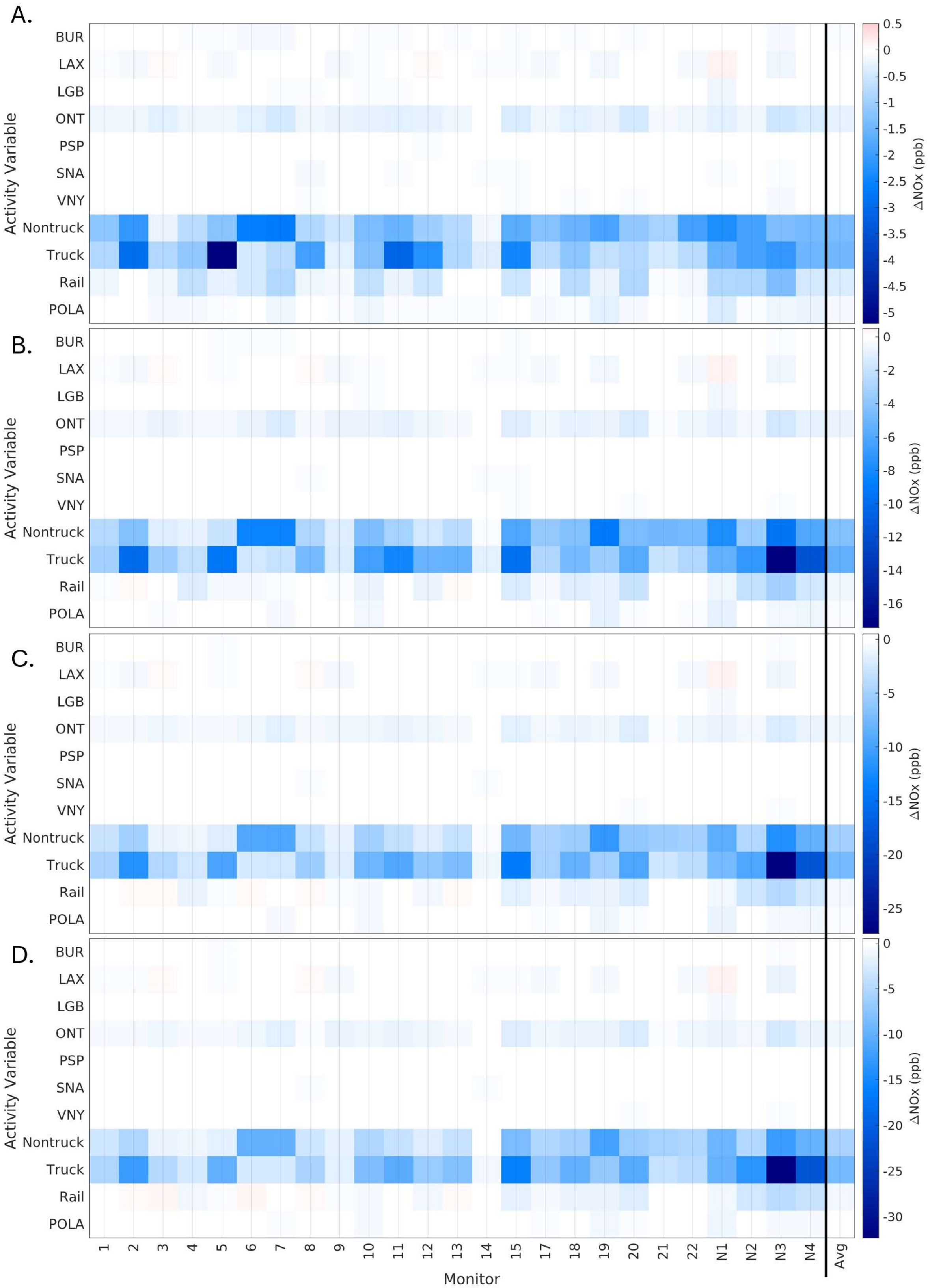

Figure S6. NO_x concentration impacts by activity variable for 6-8 am PT under a) 10%, b) 30%, c) 40%, and d) 50% decreased activity scenarios.

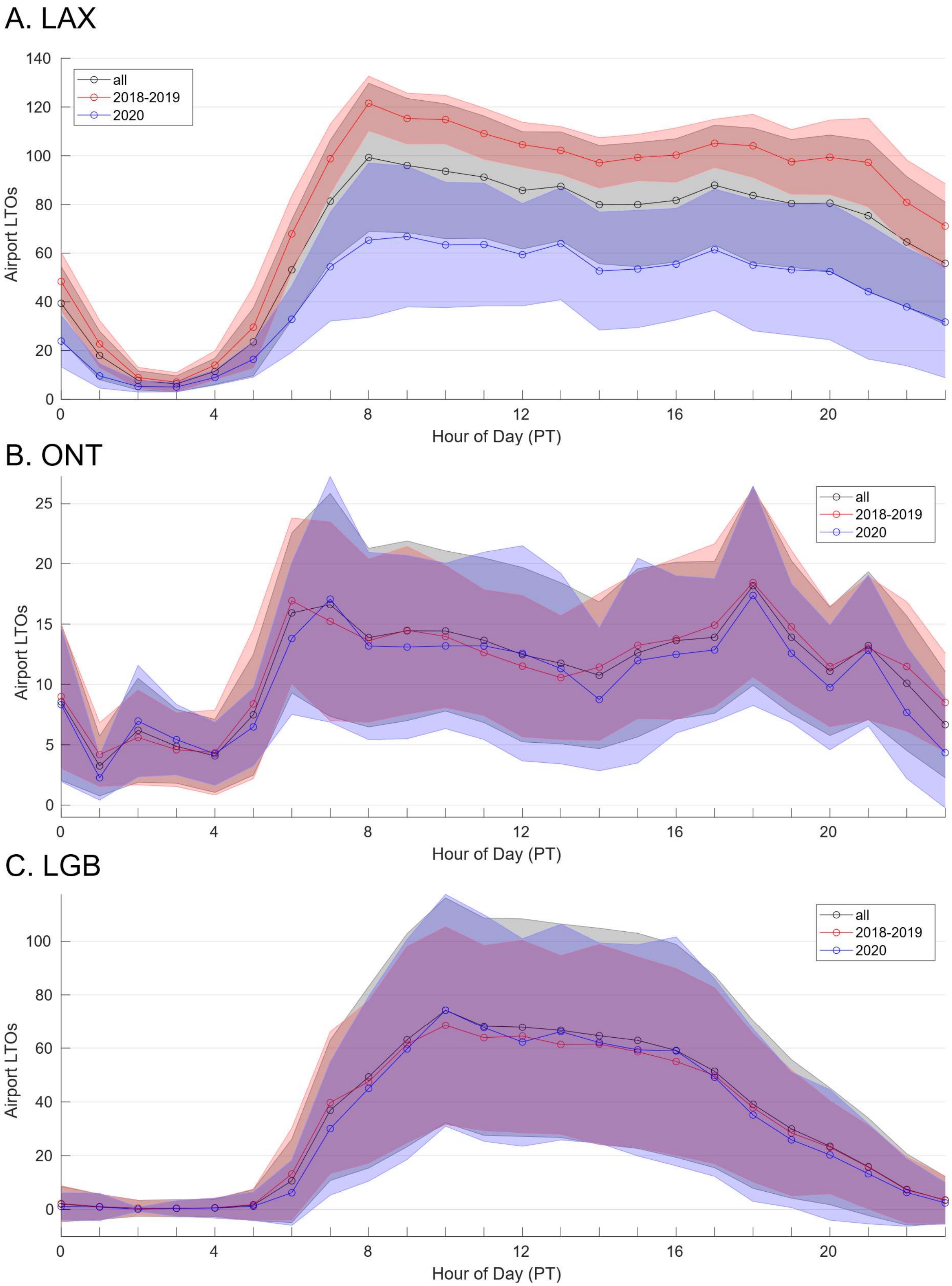

Figure S7. Diurnal profiles of aircraft landings and takeoffs (LTOs) at a) LAX, b) ONT, and c) LGB for the 2018-2021 (black), 2018-2019 (red), and 2020 (blue). Shaded areas are standard deviations.

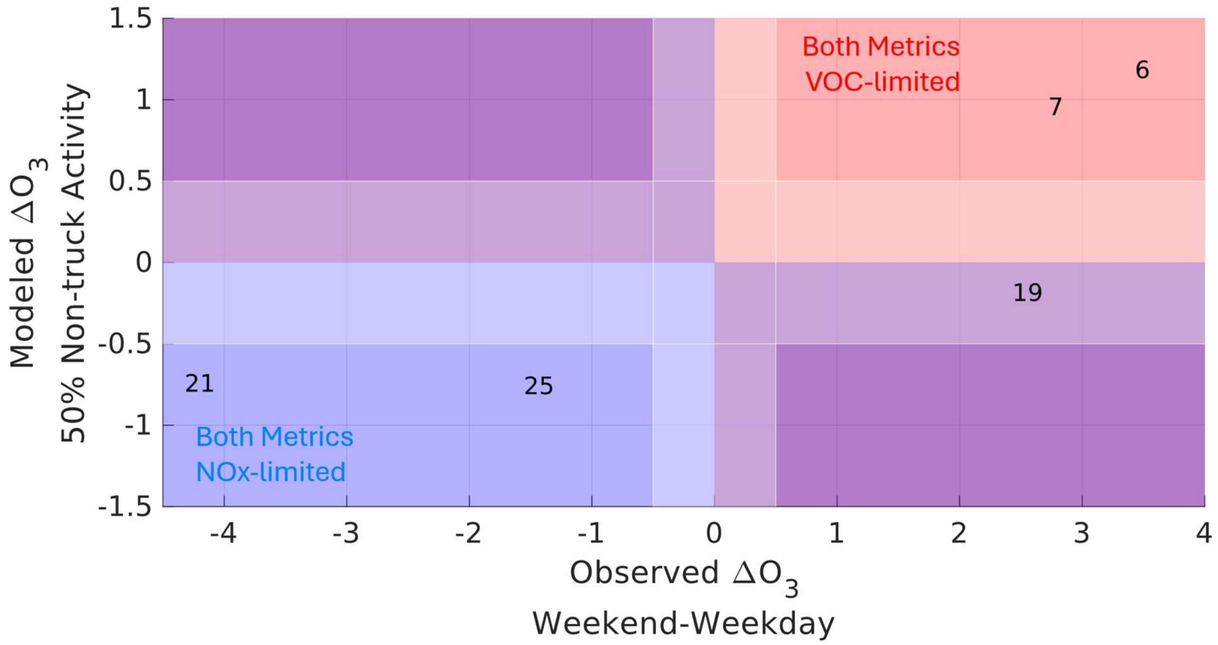

Figure S8. Ozone regime analysis using non-truck traffic reductions for monitors with average freight contribution less than 40% (monitors 6, 7, and 21). Also included are monitors that show opposite regime for freight analysis.
